# The impact of conservation-driven translocations on blood parasite prevalence in the Seychelles warbler

**DOI:** 10.1038/srep29596

**Published:** 2016-07-13

**Authors:** Eleanor A. Fairfield, Kimberly Hutchings, Danielle L. Gilroy, Sjouke A. Kingma, Terry Burke, Jan Komdeur, David S. Richardson

**Affiliations:** 1 School of Biological Sciences, University of East Anglia, Norwich Research Park, Norwich, Norfolk, NR4 7TJ, UK; 2 Behavioural and Physiological Ecology, Groningen Institute for Evolutionary Life Sciences, University of Groningen, PO Box 11103, 9700 CC Groningen, The Netherlands; 3 Department of Animal and Plant Sciences, University of Sheffield, Sheffield, S10 2TN, UK; 4 Nature Seychelles, Centre for Environment and Education, The Sanctuary, PO Box 1310, Roche Caiman, Victoria, Mahé, Republic of Seychelles

## Abstract

Introduced populations often lose the parasites they carried in their native range, but little is known about which processes may cause parasite loss during host movement. Conservation-driven translocations could provide an opportunity to identify the mechanisms involved. Using 3,888 blood samples collected over 22 years, we investigated parasite prevalence in populations of Seychelles warblers (*Acrocephalus sechellensis*) after individuals were translocated from Cousin Island to four new islands (Aride, Cousine, Denis and Frégate). Only a single parasite (*Haemoproteus nucleocondensus*) was detected on Cousin (prevalence = 52%). This parasite persisted on Cousine (prevalence = 41%), but no infection was found in individuals hatched on Aride, Denis or Frégate. It is not known whether the parasite ever arrived on Aride, but it has not been detected there despite 20 years of post-translocation sampling. We confirmed that individuals translocated to Denis and Frégate were infected, with initial prevalence similar to Cousin. Over time, prevalence decreased on Denis and Frégate until the parasite was not found on Denis two years after translocation, and was approaching zero prevalence on Frégate. The loss (Denis) or decline (Frégate) of *H. nucleocondensus*, despite successful establishment of infected hosts, must be due to factors affecting parasite transmission on these islands.

Across a wide range of host taxa, parasite diversity and abundance is lower in introduced compared to native populations^1^,^2^. This loss of parasites can have consequences for the establishment and long-term success of host populations that colonise new areas^3^,^4^. Understanding the processes that drive parasite loss in introduced populations is therefore important for protecting biodiversity, regardless of whether the aim is to reduce the impact of an invasive species, or to help conserve newly established populations of an endangered one. However, little is known about the relative importance of the different processes that are hypothesised to cause parasite loss during host introduction^5^.

There are three main processes that determine whether a parasite will successfully colonise a new area following introduction of its host^1^,^5^. First, introduced populations are usually derived from a subset of the original source population. If parasites are patchily distributed among the native host population, then founding individuals may not carry parasites with them simply as a result of biased sampling (missing the boat)^1^,^5^. Second, if infected hosts die following introduction and fail to persist, then their parasites will also fail (sinking with the boat)^5^. Third, in cases where infected founding individuals survive to establish a population, parasites could fail to persist owing to insufficient, or a complete lack of, transmission among hosts (lost overboard)^5^. Parasite transmission can be disrupted due to a low abundance or density of hosts, or, in cases where parasites have complex life cycles and require more than one host, an absence of an intermediate host or vector^1^,^5^.

It has been proposed that parasite loss can explain the success of invasive species (enemy release hypothesis)^3^,^6^,^7^. Consequently, numerous studies have investigated patterns of parasite loss in introduced exotic species^1^,^2^,^8^,^9^. However, surprisingly few studies have considered species that have expanded their range naturally (but see refs 10, 11, 12), and fewer still have reported on parasite loss occurring when hosts are deliberately translocated to establish new populations in the wild or captivity for conservation purposes^13^. Translocations are increasingly used as a conservation tool to create new populations of a species^14^. Due to the devastating impacts that introduced parasites can have on endangered species^15^,^16^, conservationists have been urged to carefully consider the risks of accidently transmitting parasites to new areas and/or exposing a translocated population to new infections^17^. While there is evidence that conservation-driven translocations can result in parasite loss within new host populations^13^, as far as we are aware, no study has tried to identify the mechanisms involved.

A major benefit of using translocations to explore mechanisms of parasite loss following host introduction is that it is possible to identify founding individuals, determine where they were sourced, and potentially which parasites they carried with them. Furthermore, the process of parasite loss can be tracked immediately following translocation. This is something that has not been possible in studies of invasive exotic species^1^ because the establishment phase of introductions is rarely observed^7^. The reasons that parasite loss may occur in translocated populations are predicted to be the same as for invasive species. However, when dealing with endangered species, the number of individuals that can be translocated is inherently small, so that any effects due to small population sizes may be exacerbated, which increases the likelihood of parasites being lost^1^.

In this study, we assess evidence of parasite loss in the Seychelles warbler (*Acrocephalus sechellensis*) after known numbers of between 29 and 59 individuals were successfully translocated from the last remaining population on Cousin Island to four new islands (Aride, Cousine, Denis and Frégate) over a period of 25 years^18^,^19^,^20^. Seychelles warblers harbour remarkably few parasites; only one strain (GRW1)^21^ of the Haemosporidian blood parasite *Haemoproteus nucleocondensus* (formerly known as *H. payevskyi*^22^), has been detected in the population on Cousin, and no other blood or gastrointestinal parasites have been found despite extensive screening^23^,^24^. *Haemoproteus* parasites (a sister genus to the *Plasmodium* genus, which includes the parasites that cause malaria) are a group of insect-vector-borne protozoan parasites. *Haemoproteus* parasites have severely impacted populations of a number of island endemic birds^16^, and infection with *H. nucleocondensus* can impose physiological costs in the Seychelles warbler^24^,^25^. On Cousin, Seychelles warblers have been caught and sampled each year since 1994. Intensive sampling of the new populations, established through translocation, has also been conducted (Aride 1993–2013; Cousine 1997–2011; Denis 2004–2013; Frégate 2011–2013), with repeated sampling of the same individuals and multiple generations sampled over time. In the cases of Aride and Cousine, routine blood sampling began five and three years after translocation respectively, but for Denis and Frégate founders were sampled before and after translocation. These longitudinal data provide us with a unique opportunity to investigate patterns of *H. nucleocondensus* prevalence in newly established populations after repeated translocation events.

Specifically, we aim to (1) compare the prevalence of *H. nucleocondensus* in Seychelles warblers on Cousin Island to that on the four newly-established populations to assess whether there has been a loss of infection as a result of the translocations. (2) Describe and compare the pattern of *H. nucleocondensus* prevalence across years for each island to determine which of the three alternative processes (‘missing the boat’, ‘sinking with the boat’, or ‘lost overboard’) is the most likely cause of any observed parasite loss.

## Results

**Figure 1:**
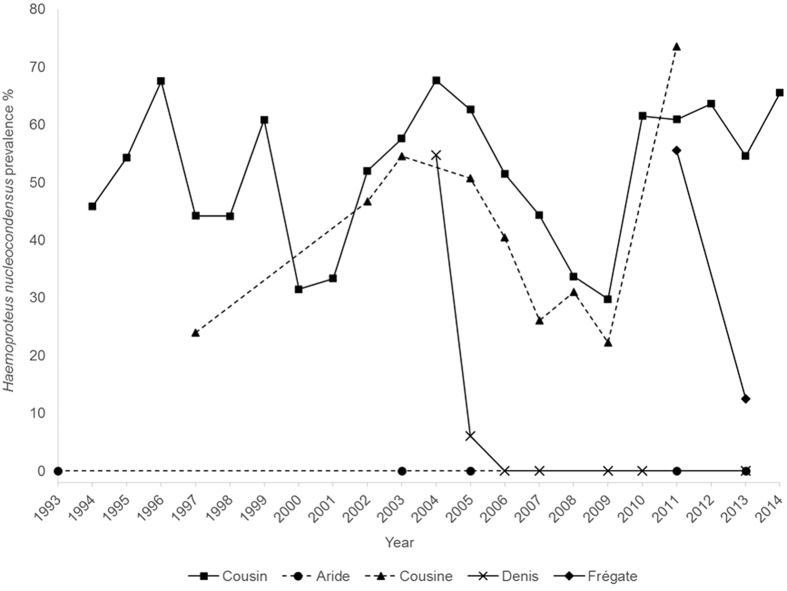
Mean GRW1 *Haemoproteus nucleocondensus* prevalence as a percentage of the individuals infected for each island and year sampled from 1993 until 2014.

Among the newly established populations, *Haemoproteus* infection has only persisted on Cousine (Fig. 1), and DNA sequencing confirmed that this population was infected with the same strain (GRW1^21^) of *Haemoproteus nucleocondensus* previously and consistently detected in the source population of Cousin^23^. Infection prevalence fluctuated between 30–68% (mean = 52%) on Cousin and between 24–74% (mean = 41%) on Cousine. Overall, infection probability did not differ between Cousin and Cousine (*Z* = 1.67, *P* = 0.10), and there was a significant correlation in *H. nucleocondensus* prevalence between these islands across years (*r* = 0.84, *P* = 0.005). Juveniles were more likely to be infected than adults (72% and 32% prevalence in juveniles and adults respectively, *Z* = −14.81, *P* < 0.001), and while there was a tendency for males to be infected more often than females, this difference was not significant (53% and 47% prevalence in males and females respectively, *Z* = 1.60, *P* = 0.07; Table 1).

None of the Seychelles warblers that were born on Aride (*n* = 136), Denis (*n* = 367) and Frégate (*n* = 13) were infected with *H. nucleocondensus*. Sampling on Aride began five years after translocation, and only five original founders were alive and sampled at the earliest sampling date; none were found to be infected. It is not known whether the individuals translocated to Aride were never infected with *H. nucleocondensus*, or if they either died or eliminated the infection.

Individuals translocated to Denis and Frégate were sampled before and after translocation. Among the individuals that were translocated to Denis, 55% were infected with *H. nucleocondensus* before they were translocated in 2004 (*n* = 42), and thereafter prevalence declined until none of the surviving founding individuals were infected from 2006 onwards (*n* = 17). Eight Seychelles warblers that were infected with *H. nucleocondensus* before they were translocated to Denis were found to have lost the infection when the same individuals were re-screened one to six years post-translocation. Of the individuals translocated to Frégate that were sampled before translocation (*n* = 36), 56% were infected with *H. nucleocondensus*. Two years later, two out of the three founding individuals that we re-sampled still had detectable infection (67%).

## Discussion

Seychelles warblers were translocated to four previously uninhabited islands from a source population (Cousin Island) where a single parasite has been identified^23^ (the GRW1^21^ strain of *Haemoproteus nucleocondensus;* a Haemosporidian blood parasite) and occurs at an average prevalence of 52%. A similar prevalence of *H. nucleocondensus* has persisted on Cousine (41%), but no infection was found in any of the new birds that hatched on Aride, Denis and Frégate. In the case of Aride, we do not know whether *H. nucleocondensus* was ever introduced to the island with the founders, but our pre- and post-translocation sampling of individuals translocated to Denis and Frégate allowed us to confirm that the initial prevalence of *H. nucleocondensus* in the founding individuals of these islands was similar to that on Cousin. Infection prevalence gradually declined to extinction among the birds translocated to Denis, but among the original individuals translocated to Frégate, infection prevalence nominally rose from 45% immediately after translocation to 67% two years later. However, only three surviving founders were re-sampled (Table 2), and the two individuals that carried *H. nucleocondensus* were infected before they were taken to Frégate. With this in mind, and the fact that *H. nucleocondensus* has not been transmitted to any of the new birds hatched on Frégate, it is likely that the *H. nucleocondensus* parasite will also decline to extinction on Frégate.

There are three main processes that determine whether or not a parasite will successfully colonise a new area following the introduction of its host^1^,^5^. First, the founders may simply not have carried any parasites with them (‘missing the boat’), perhaps due to low, or aggregated patterns of, infection in the original population^1^,^5^. In our study, we were able to confirm that *H. nucleocondensus* was introduced to at least two of the new islands (Denis and Frégate), and it is reasonable to assume that the non-sampled founders taken to Cousine and Aride were also infected. There is virtually no migration of Seychelles warblers between islands^26^, and so it is unlikely that *H. nucleocondensus* reached Cousine after translocation through the natural dispersal of Seychelles warblers. It is possible that there has been dispersal of infected vectors between Cousin and Cousine, and some species of *Culicoides* biting midges, one of the known vectors of *Haemoproteus* parasites, can be dispersed hundreds of kilometres by the wind^27^. However, given that the founders of Aride and Cousine were taken from a source population where *H. nucleocondensus* prevalence averages 52%, and all individuals were chosen blind in regards to their infection status, it is very unlikely that only uninfected individuals were caught and transferred to those islands.

Second, infected hosts may fail to survive and persist following introduction, in which case the parasite would also fail to establish (‘sinking with the boat’), assuming there are no alternative hosts in the new range^5^. Importantly, by re-sampling a proportion of the same individuals translocated to Denis and Frégate before and after translocation (Table 2), we were able to show that infected hosts can survive following translocation. On each of Denis and Frégate, two Seychelles warblers that were infected with *H. nucleocondensus* before they were translocated were still infected and alive one and two years after translocation respectively. On Denis, eight Seychelles warblers that were originally infected with *H. nucleocondensus* were shown to have lost the infection (possibly through individual clearance of the infection and a subsequent lack of re-infection) when the same individuals were re-screened from one to six years following their introduction. Therefore, since infected birds either survived despite infection, or cleared the disease, the loss (Denis) or decline (Frégate) of *H. nucleocondensus* on these islands was not solely due to infected hosts dying after arrival. If infected birds were also taken to Aride as we predict (see above), there is no reason to assume they would be any less likely to survive than the infected birds taken to Denis and Frégate. Furthermore, *Haemoproteus* parasites are mostly host specific^28^. The GRW1 strain of *H. nucleocondensus* has also been detected in the closely related great reed warbler (*Acrocephalus arundinaceus*)^29^. However, within the Seychelles, GRW1 has not been found in any of the other seven passerine birds screened for Haemosporidian blood parasites^30^ (See Supplementary Table S1 for additional unpublished data), and the three Seychelles passerine species that do carry any type of Haemosporidian blood parasite are infected by a different genus (*Plasmodium*)^30^. The screened species represent most of the passerine fauna of the Seychelles, and so this suggests that the presence and/or transmission of GRW1 in the Seychelles warblers are not influenced by the presence of other avian hosts.

We know from observations on Denis and Frégate that *H. nucleocondensus* did not ‘miss the boat’ or ‘sink with the boat’. Therefore, another factor must have prevented transmission of this parasite to any new birds born on Aride, Denis and Frégate, and driven the parasite, but not the host, to decline in overall prevalence (Frégate) or go extinct (Denis) despite being introduced to these islands. The ‘lost overboard’ hypothesis predicts a parasite will fail to persist in a new host population if parasite transmission is insufficient, or completely lacking, in the new range^1^,^5^. One possible reason for this is that the founding populations were too small for any introduced infection to be transmitted from one host to another, and therefore to persist^7^. However, in the Seychelles warbler, *H. nucleocondensus* did persist after the translocation to Cousine, which was founded by an equally small number of individuals as Aride (*n* = 29), and half the number of founders moved to Denis (*n* = 58) and Frégate (*n* = 59). In addition, the source population on Cousin experienced a bottleneck in the 1960s^31^, which saw the number of Seychelles warblers fall below 50 individuals for several decades^32^ without the infection being lost.

Parasite transmission can also be disrupted if the host population density is too low^1^. Aride and Denis are considerably larger (0.68 km^2^ and 1.42 km^2^, respectively) than Cousin and Cousine (0.29 km^2^ and 0.25 km^2^, respectively). If larger island size results in lower host densities, and therefore less effective parasite transmission, then this could explain the loss of *H. nucleocondensus* on Aride and Denis. However, population monitoring shows that while overall island densities are low on Aride and Denis^33^, the initial habitat used for territories was restricted to the insect- and vegetation-rich release sites, and Seychelles warbler density was very high in these areas of the islands^18^,^19^,^20^,^34^. It therefore seems unlikely that host density would have been the driver of *H. nucleocondensus* loss in the Seychelles warbler.

The remaining reason for parasites being ‘lost overboard’ in newly established populations is a lack of appropriate parasite vectors. *Haemoproteus* parasites are transmitted through biting insects, and the abundance and spatial distribution of these vectors is regarded as an important factor in the incidence of insect-vector-borne diseases^35^. In the Seychelles warbler, the insect vector responsible for transmitting *H. nucleocondensus* on Cousin may be present on the nearby island of Cousine but absent on the more distant (and isolated) islands of Aride, Denis and Frégate. We do not know the specific vector that transmits the GRW1 strain of *H. nucleocondensus*, but, generally speaking, *Haemoproteus* parasites are transmitted by biting midges (Ceratopogonidae) and louse flies (Hippoboscidae)^36^. During each translocation, Seychelles warblers were released in wet areas with dense vegetation where insects were most abundant on that particular island^18^,^19^,^20^. If present, we believe that any insect *Haemoproteus* vectors would be most likely to occur in these same areas. Therefore, it is possible that Seychelles warblers on Aride, Denis and Frégate were not exposed to the appropriate insect vectors because they simply do not exist there, and that the absence of suitable insect vectors prevented the transmission of *H. nucleocondensus* to new birds, and led to the overall decline (Frégate) and extinction (Aride and Denis) of the parasite on these islands.

It is unclear why insect vectors could be absent from individual islands within an archipelago, though it could be linked to the past clearance of native habitat for coconut (*Cocos nucifera*) plantations^20^, and the use of insecticides. Both Frégate and Denis were large plantations that have only recently started to be restored. In addition, Frégate and Denis are further away and more isolated from the larger main islands compared to Cousin and Cousine, which may have prevented re-colonisation by these insect vectors. Therefore, although Seychelles warblers were released in the most insect-rich areas for each island, past differences in habitat and pest management may have resulted in differences in insect abundance and diversity between islands. Surveys of possible GRW1 vectors on each island are now needed to verify our speculation that the appropriate vectors are absent on Aride, Denis and Frégate, and, if so, to understand why they only exist on Cousin and Cousine (where prevalence of *H. nucleocondensus* across years is strongly correlated). Such surveys will enable us to confirm exactly why the GRW1 strain of *H. nucleocondensus* has persisted, declined, or become extinct during the Seychelles warbler translocations.

Understanding the processes that drive parasite loss in translocated populations could aid conservation of the target species, but a consideration of the possible pros and cons of parasite loss in new host populations is necessary. The translocation of Seychelles warblers to new islands has been successful in all four attempted cases^18^,^19^,^20^, but it seems unlikely that the loss of *H. nucleocondensus* has contributed significantly to this success. Although *H. nucleocondensus* is thought to carry costs for the Seychelles warbler^24^,^25^, its high prevalence did not prevent the population on Cousin from rapidly recovering to carrying capacity after re-forestation of the island^18^. Similarly, the infected population on Cousine successfully established and rapidly reached carrying capacity^19^. Therefore, *H. nucleocondensus* does not seem to constrain these Seychelles warbler populations, though it is possible that parasite absence may be particularly important during the early phases of introduction and may contribute to high rates of population growth^10^,^37^. The negative effects of *H. nucleocondensus* in Seychelles warblers are more pronounced in early life, with infection thought to reduce survival in juveniles^24^. Our results show that *H. nucleocondensus* never gets transmitted to birds born on Denis, Aride and Frégate. Consequently, with potentially fewer birds dying before reaching adulthood, the absence of *H. nucleocondensus* may have, to some extent, contributed to the rapid population growth that we have observed on the new islands^19^,^33^.

There could, however, be negative long-term effects of parasite loss in translocated populations. Populations lacking parasites may experience temporary benefits in the short term^38^, but could be more susceptible to new parasites in the future owing to a lack of host-parasite co-evolutionary history, or a loss of genetic diversity at important pathogen-defence loci such as the Major Histocompatibility complex (MHC)^4^. While overall parasite species richness tends to be lower in introduced populations, new parasites are commonly gained over time^1^,^2^,^8^. Novel parasites have had a damaging impact on island vertebrate species^15^, and introduced Haemosporidian parasites specifically have been implicated in the decline and extinction of several island endemic birds^16^. In the Seychelles warbler, the loss of *H. nucleocondensus* on Aride, Denis and Frégate means that these populations will have no identified parasites^23^, the absence of which would halt any host-parasite coevolution. It is reassuring that there has been little loss of MHC diversity in the new populations as a result of the translocations^39^, although we do not know if the loss of some rare alleles could be significant in the long term if these populations were ever exposed to a novel parasite.

We have demonstrated that closely monitored conservation-driven translocations may provide excellent systems and data to help us unravel the processes that cause parasite loss in new populations. This information should aid our ability to predict the fate of hosts and their parasites following host movement. Such information is necessary to manage the impact of invasive host species, or to mitigate the effects of parasites on endangered host species, when colonising new areas. From a conservation perspective, whether the loss of parasites in endangered species will have positive or negative consequences may not be immediately clear, and may depend upon the timeframe one considers. However, in situations where populations of endangered species are currently suffering from the impacts of parasites, parasite loss through translocation could be a useful consideration for conservation.

## Methods

### Study species and populations

The Seychelles warbler is a small insectivorous passerine endemic to the Seychelles in the western Indian Ocean^40^. The species underwent a severe population bottleneck during the early 1900 s^32^, and by the 1960 s the population size was reported to be less than 30 individuals confined to the island of Cousin^31^ (4°20′S, 55°40′E, 0.29 km^2^). Conservation management on Cousin has since facilitated the population’s recovery and it has remained stable at *ca.*320 birds since 1982^18^,^33^. Inter-island dispersal is extremely rare (0.1%)^26^ and, in order to further safeguard the Seychelles warbler and allow population expansion, translocations of individuals from Cousin to four other islands have been carried out. Twenty-nine birds were translocated to Aride (4°12′S, 55°40′E, 0.68 km^2^, 9 km from Cousin) in 1988^18^, 29 birds to Cousine (4°21′S, 55°39′E, 0.25 km^2^, 2.5 km from Cousin) in 1990^18^, 58 birds to Denis (3°48′S, 55°40′E, 1.42 km^2^, 63 km from Cousin) in 2004^19^ and 59 birds to Frégate (4°35′S, 55°56′E, 2.19 km^2^, 45 km from Cousin) in 2011^20^. The founders for each translocation were caught randomly across Cousin Island and blind with respect to *H. nucleocondensus* infection status, but care was given to avoid very old birds and those in poor condition. Age was resolved based on eye colour^41^ and sex was determined using a molecular method^42^.

### Data collection and parasite screening

The Seychelles Bureau of Standards and the Department of Environment gave permission for sampling and fieldwork, and all work was approved by the University of East Anglia animal welfare and ethical review body and carried out in accordance with local ethical regulations and agreements. Data were collected during the main breeding season (July–September) and, in some years, during the smaller breeding season (December–February) in the period 1993–2014. Not all islands were visited every year with the exception of Cousin (Table 2). Birds were caught using mist nets and ringed with a unique combination of colour and British Trust for Ornithology rings. Blood samples (~25 μl) were taken by brachial venipuncture and stored in absolute ethanol. Following DNA extraction using a salt extraction technique^43^, infection by Haemosporidian blood parasites was screened for using a nested PCR^44^. This method consists of 20 cycles using the primers HaemNF1 and HaemNR3, followed by a final amplification of 40 cycles using the primers HaemF and HaemR2, which target a 479-bp section of the cytochrome *b* gene of *Haemoproteus* and *Plasmodium* parasites. PCR-based methods may underestimate the prevalence of Haemosporidian blood parasites because parasite DNA can occur at low concentrations in the blood. Therefore, to reduce the occurrence of false negatives, all samples were screened for *Haemoproteus* and *Plasmodium* parasites twice. Samples were recorded as being infected if they tested positive in either of the two independent PCRs, and 87% were consistently scored as positive or negative across these two runs.

The average annual percentage of the total population screened for Haemosporidian blood parasites was 42% on Cousin, 3% on Aride, and 28% on Cousine (Table 2). Of the birds hatched on Denis and Frégate, an annual average of 36% and 16% of the total population was screened, respectively. Among the founders, 19 of the birds translocated to Denis (33%) and 24 birds translocated to Frégate (41%) were screened less than six months prior to translocation. However, we avoided blood sampling birds immediately before translocation to minimise any stress. If individuals that were translocated to Denis and Frégate had not been blood sampled during this period, samples were screened from within the two years preceding translocation (an additional 23 birds from Denis and 12 birds from Frégate). The founders of Aride and Cousine were not screened before translocation because we did not take blood samples until five and three years after translocation, respectively.

Previous analysis has shown that Seychelles warblers on Cousin are host to just one strain (GRW1^21^) of *H. nucleocondensus*^23^ (formerly *H. payevskyi*^22^). Ten further positive samples taken over the years from birds inhabiting Cousine were sequenced to confirm the strain of Haemosporidian detected in this population using the Big Dye terminator kit v. 3.1 (Applied Biosystems) following Hutchings^23^. Sequences were aligned against sequences from the National Centre for Biotechnology Information (NCBI) GenBank database using MEGA^45^.

### Statistical analyses

In years where data were available for each island, differences in *H. nucleocondensus* prevalence between Cousin and Cousine islands (*n* = 1,978) were assessed using a generalized linear mixed effects model fitted in R 3.1.1^46^ using the lme4 package^47^, with infection expressed as a binomial response variable. Our dataset included juveniles (<1 year) and adults (>1 year). Nestling samples were excluded because the length of time for which Seychelles warbler nestlings are in the nest^48^ and the prepatent period for *Haemoproteus*^36^ only overlaps for a couple of days. Therefore, unless a nestling is infected immediately after hatching, infection is unlikely to be detected at this early life stage. Data from the major and minor breeding seasons were combined because individual infection prevalence does not differ between seasons^23^. Sex and age class were included as covariates because of the previously observed association between these variables and *H. nucleocondensus* infection in the Seychelles warbler^24^. Year and individual identity were included as random factors to control for multiple measurements of the same bird across years. We compared *H. nucleocondensus* prevalence across years on Cousin and the neighbouring island of Cousine using a Pearson correlation analysis (Spearman’s *r*).

## Additional Information

**How to cite this article**: Fairfield, E. A. *et al*. The impact of conservation-driven translocations on blood parasite prevalence in the Seychelles warbler. *Sci. Rep.* **6**, 29596; doi: 10.1038/srep29596 (2016).

## Supplementary Material

Supplementary Information

## Figures and Tables

**Table 1:** A generalized linear mixed model comparing GRW1 *Haemoproteus nucleocondensus* prevalence in Seychelles warblers between Cousin and Cousine islands.

*Variables*	Estimate	SE	*Z*	*P*
Island	0.220	0.133	1.665	0.100
Age	−1.818	0.123	−14.810	2 × 10^−16^
Sex	0.173	0.109	1.596	0.066

**Table 2:** Number of samples screened for Haemosporidian parasites (*Haemoproteus* and *Plasmodium*) in each Seychelles warbler population in each year since blood sampling started in 1993, with the percentage of the total island population screened in that year in parentheses.

Year	Cousin		Cousine		Aride		Translocated to Denis		Born on Denis		Translocated to Frégate		Born on Frégate	
1993	—	—	—	—	29	(7%)	—	—	—	—	—	—	—	—
1994	85	(28%)	—	—	—	—	—	—	—	—	—	—	—	—
1995	116	(36%)	—	—	—	—	—	—	—	—	—	—	—	—
1996	37	(11%)	—	—	—	—	—	—	—	—	—	—	—	—
1997	138	(48%)	25	(18%)	—	—	—	—	—	—	—	—	—	—
1998	111	(38%)	—	—	—	—	—	—	—	—	—	—	—	—
1999	92	(31%)	—	—	—	—	—	—	—	—	—	—	—	—
2000	54	(17%)	—	—	—	—	—	—	—	—	—	—	—	—
2001	15	(5%)	—	—	—	—	—	—	—	—	—	—	—	—
2002	148	(45%)	30	(18%)	—	—	—	—	—	—	—	—	—	—
2003	165	(51%)	11	(7%)	9	(1%)	—	—	—	—	—	—	—	—
2004	130	(39%)	—	—	—	—	42	(72%)	—	—	—	—	—	—
2005	198	(61%)	71	(43%)	37	(2%)	6	(8%)	27	(36%)	—	—	—	—
2006	132	(39%)	47	(29%)	—	—	5	(5%)	49	(44%)	—	—	—	—
2007	194	(53%)	119	(57%)	—	—	—	—	28	(22%)	—	—	—	—
2008	187	(56%)	116	(55%)	—	—	—	—	—	—	—	—	—	—
2009	198	(63%)	9	(4%)	—	—	7	(4%)	90	(45%)	—	—	—	—
2010	104	(33%)	—	—	—	—	3	(1%)	90	(43%)	—	—	—	—
2011	156	(50%)	34	(16%)	28	(2%)	—	—	—	—	36	(61%)	—	—
2012	165	(54%)	—	—	—	—	—	—	—	—	—	—	—	—
2013	174	(59%)	—	—	33	(2%)	—	—	83	(28%)	3	(4%)	13	(16%)
2014	209	(62%)	—	—	—	—	—	—	—	—	—	—	—	—
