# Supplementary material for: The impact of conservation-driven translocations on blood parasite prevalence in the Seychelles warbler
Source: Sci Rep. 2016 Jul 13;6:29596. doi: 10.1038/srep29596 (PMC4942767; doi:10.1038/srep29596)
Supplement: Supplementary Information [file srep29596-s1.pdf]

Supplementary information:

The impact of conservation-driven translocations on blood parasite prevalence in the Seychelles warbler

Eleanor A. Fairfield[1], Kimberly Hutchings[1, 2], Danielle L. Gilroy[1], Sjouke A. Kingma[2], Terry Burke[3], Jan Komdeur[2] and David S. Richardson[1, 4*].

1. School of Biological Sciences, University of East Anglia, Norwich Research Park, Norwich, Norfolk, NR4 7TJ, UK.
2. Behavioural and Physiological Ecology, Groningen Institute for Evolutionary Life Sciences, University of Groningen, PO Box 11103, 9700 CC Groningen, The Netherlands.
3. Department of Animal and Plant Sciences, University of Sheffield, Sheffield, S10 2TN, UK.
4. Nature Seychelles, Centre for Environment and Education, The Sanctuary, PO Box 1310, Roche Caiman, Victoria, Mahé, Republic of Seychelles.

Corresponding author: David Richardson: david.richardson@uea.ac.uk

Supplementary Table S1: Passerine birds screened for avian malaria parasites (*Haemoproteus* and *Plasmodium*) across islands of the Seychelles archipelago. No samples tested positive.

Island	*Species*	*n*
Conception	Seychelles white-eye (*Zosterops modestus*)	27
Cousin	Seychelles sunbird (*Cinnyris dussumieri*)	45
Cousine	Seychelles sunbird (*Cinnyris dussumieri*)	5
	Madagascar fody (*Foudia madagascariensis*)	12
Denis	Madagascar fody (*Foudia madagascariensis*)	4
Frégate	Seychelles sunbird (*Cinnyris dussumieri*)	5
	Seychelles magpie-robin (*Copsychus sechellarum*)	16
	Madagascar fody (*Foudia madagascariensis*)	6
	Seychelles white-eye (*Zosterops modestus*)	5
La Digue	common myna (*Acridotheres tristis*)	19
	Seychelles bulbul (*Hypsipetes crassirostris*)	10
Mahé	common myna (*Acridotheres tristis*)	55
	Seychelles bulbul (*Hypsipetes crassirostris*)	73
Praslin	common myna (*Acridotheres tristis*)	30
	Seychelles bulbul (*Hypsipetes crassirostris*)	33
